# Plastic Pollution and Child Health: A Narrative Review of Micro- and Nanoplastics, Additives, and Developmental Risks

**DOI:** 10.3390/jcm14238399

**Published:** 2025-11-27

**Authors:** Nicola Principi, Alberto Argentiero, Beatrice Rita Campana, Hajrie Seferi, Elena Cinti, Susanna Esposito

**Affiliations:** 1 Università degli Studi di Milano, 20122 Milan, Italy; nicola.principi@unimi.it; 2 Pediatric Clinic, Department of Medicine and Surgery, University of Parma, 43126 Parma, Italy; alberto.argentiero@unipr.it (A.A.); beatricerita.campana@unipr.it (B.R.C.); hajrie.seferi@unipr.it (H.S.); elena.cinti@unipr.it (E.C.)

**Keywords:** microplastics, nanoplastics, plastic additives, bisphenols, phthalates, endocrine disruption

## Abstract

Plastic production and subsequent environmental contamination have increased substantially in recent decades, resulting in pervasive human exposure to microplastics (MPs), nanoplastics (NPs), and plastic-associated additives such as bisphenols and phthalates. These substances are known to induce toxic effects via multiple biological mechanisms, including oxidative stress, inflammation, apoptosis, immune system disruption, and genotoxicity. While exceptions exist, current research generally indicates that these exposures may adversely affect fertility. Notably, children constitute the most vulnerable demographic due to behavioral tendencies, higher intake-to-body-weight ratios, underdeveloped detoxification systems, and critical developmental periods of susceptibility. Evidence demonstrates that exposure commences in utero, with MPs, NPs, and additives identified in placental tissue, amniotic fluid, cord blood, and meconium—factors associated with impaired fetal growth and reduced gestational duration. After birth, additional exposure occurs through diet, inhalation, household dust, feeding equipment, toys, and consumer products. Experimental and epidemiological studies suggest that plastics may adversely affect multiple physiological systems. Reported outcomes include altered pubertal development, reduced fertility, neurodevelopmental abnormalities, respiratory diseases such as asthma, and increased risks of metabolic disorders, including obesity and insulin resistance. However, substantial knowledge gaps remain: the relative toxicity of different polymers and additives, dose–response relationships, critical exposure periods, and long-term consequences are not yet fully defined. Given growing concern and mounting evidence of harm, precautionary measures are warranted. Reducing nonessential plastic use, strengthening regulatory actions, improving product labeling, and promoting public awareness are urgent priorities, particularly in vulnerable and resource-limited communities. Further mechanistic studies and longitudinal human research are essential to clarify health risks, guide safer material substitutions, and inform evidence-based policies aimed at protecting children from avoidable plastic-related toxicity.

## 1. Background

Plastics have progressively supplanted traditional materials such as glass and metals in numerous industrial sectors owing to their exceptional versatility, durability, lightweight nature, and ease of molding and mass production [1]. As a result, plastics have become an essential component of modern society, finding extensive applications in packaging, construction, automotive manufacturing, electronics, consumer goods, textiles, healthcare, agriculture, and food production. Their broad functionality, low cost, and adaptability to diverse environments have significantly contributed to their widespread adoption. Current estimates indicate that global plastic consumption will continue to grow at an unprecedented rate, potentially reaching 800 million metric tons by 2050, with cumulative plastic stocks rising to 4725 million metric tons [2]. However, this exponential increase has not been accompanied by adequate end-of-life management strategies. By 2023, only about 10% of global plastic waste was recycled, while the remainder was incinerated, sent to landfills, or inadequately managed, often escaping into natural ecosystems [3]. Such practices raise substantial environmental and public-health concerns, as incineration can release hazardous substances, including dioxins, polycyclic aromatic hydrocarbons, persistent organic pollutants, and toxic metals such as lead and mercury. At the same time, mismanaged plastics have been shown to harm wildlife, accelerate biodiversity loss, disrupt ecosystem functioning, and exacerbate global climate change [4,5,6].

In the environment, plastics are exposed to a variety of abiotic and biotic degradation processes, including abrasion, biodegradation, heat, and ultraviolet radiation, ultimately leading to their progressive fragmentation. Transport by wind, rivers, and ocean currents further accelerates this process, resulting in the formation of plastic particles classified as microplastics (MPs, <5 mm) and nanoplastics (NPs, 1–1000 nm) [7,8]. Due to their persistence, buoyancy, and resistance to natural degradation, MPs and NPs are now ubiquitous, having been detected in freshwater systems, marine environments, beaches, remote islands, deep-sea sediments, and polar regions [9]. Their extremely small size facilitates cellular uptake and biological translocation, enabling these particles to cross epithelial and endothelial barriers in both plants and animals. MPs and NPs have also been identified across multiple human exposure matrices, including food, beverages, drinking water, air, and atmospheric dust, and can come into contact with the skin, particularly through personal-care products used for exfoliation or stabilization purposes [10,11]. Beyond particle toxicity, plastics can release chemical additives such as bisphenols and phthalates—compounds intentionally incorporated to enhance flexibility, fire resistance, static prevention, and coloration. As these substances leach into surrounding environments, they further increase the toxicological burden and contribute to combined or synergistic health effects [12].

Growing scientific interest has therefore focused on the potential health implications of MPs, NPs, and plastic-associated chemicals. A substantial body of research now indicates that plastic particles and additives may exert biological effects through oxidative stress, inflammation, endocrine disruption, and genotoxicity, with possible consequences for living organisms at multiple levels [13,14]. Based on in vitro models and experimental animal studies, many human investigations have increasingly concentrated on vulnerable populations, particularly children, whose developing organ systems may be more susceptible to chemical interference. Pediatric research has explored outcomes ranging from infertility to altered pre- and postnatal development, suggesting a possible association between plastic exposure and adverse child health trajectories [15]. This growing body of evidence has prompted health authorities and governmental institutions to introduce policies and strategic plans aimed at reducing plastic production, restricting hazardous additives, and limiting environmental release [16,17]. However, at present, such conclusions remain preliminary. Critical knowledge gaps persist regarding the specific components of plastics responsible for toxicity, the mechanisms through which MPs, NPs, and additives exert their effects, and the importance of exposure timing, duration, and dose during critical developmental windows.

The growing evidence of plastic exposure during fetal life and childhood has concrete implications for clinical practice. Micro- and nanoplastics and plastic-associated additives are increasingly linked with conditions frequently encountered in pediatrics—such as fetal growth restriction, asthma, allergic disease, precocious puberty, neurodevelopmental disorders, and future infertility. Understanding plastic exposure as an emerging modifiable risk factor may assist pediatricians, obstetricians, and neonatologists in the early identification of vulnerable infants, the interpretation of clinical findings, and targeted anticipatory counseling for families. Therefore, improving knowledge on exposure pathways and associated health risks is essential to guide preventive strategies and strengthen child-health protection.

For all these reasons, further investigation is urgently needed. This study aims to provide a comprehensive overview of the existing knowledge regarding MPs, NPs, and related plastic additives, specifically examining their implications for pediatric exposure and toxicity with an emphasis on developmental health. Additionally, it underscores critical research priorities and the need for coordinated public health strategies to reduce plastic-associated risks among children.

## 2. Methods

This narrative review was conducted to summarize current evidence regarding microplastics (MPs), nanoplastics (NPs), and plastic additives, with a specific focus on pediatric exposure and health effects. A literature search was performed between January 2010 and September 2025 using the electronic databases PubMed, Scopus, Web of Science, Embase, and Google Scholar. The search strategy combined terms related to plastics and pediatric health, including: “microplastics,” “nanoplastics,” “plastic additives,” “bisphenols,” “phthalates,” “endocrine disruptors,” “children,” “pediatrics,” “prenatal exposure,” “development,” and “health effects.” Reference lists of relevant reviews and original articles were also screened to identify additional studies.

Studies were included if they: (1) reported original data from in vitro, in vivo, or human research; (2) evaluated MPs, NPs, or plastic-related chemical exposure; and (3) addressed outcomes relevant to fetal, neonatal, infant, child, or adolescent health. Narrative reviews, systematic reviews, and authoritative policy documents were considered to provide contextual background. No restrictions were placed on study design. Articles were excluded if they were unrelated to plastics, not available in English, or unrelated to pediatric or developmental outcomes.

Titles and abstracts were screened for relevance, and full texts were reviewed when eligibility was uncertain. Because this was a narrative review, no formal quality scoring system or meta-analysis was applied. Findings were synthesized qualitatively, with priority given to studies providing mechanistic insight, developmental relevance, or clinical significance. Discrepancies in interpretation were resolved by consensus among the authors.

## 3. Pediatric Exposure to Plastics

### 3.1. During Fetal Life

Exposure to plastic particles can begin as early as fetal development. The first demonstration of MPs in human placenta was reported in 2021, when Ragusa et al. analyzed six placentas from uncomplicated pregnancies and detected twelve MPs in four samples [18]. Polypropylene (PP) was the most frequently identified polymer, consistent with its widespread use in paints, adhesives, plasters, finger paints, and personal-care products. MPs were present in all sampled placental regions—five on the fetal side, four on the maternal side, and three in the chorioamniotic membrane—suggesting potential transplacental transfer to the fetus. Because only small tissue sections were examined, the true placental burden of MPs was likely underestimated. In a subsequent study, the same group reported ultrastructural alterations involving the endoplasmic reticulum and mitochondria in placental cells containing MPs, changes that may impair placental function and fetal development [19]. These findings are consistent with animal studies in which MPs and NPs accumulate in placental tissue, disrupt blood flow, and alter placental metabolism. For example, Haddadi et al. showed that pregnant rats exposed to 0.1 mg/kg of polystyrene (PS) MPs accumulated particles predominantly in the labyrinth zone, accompanied by reduced labyrinth thickness, oxidative imbalance, apoptosis, and altered ion-transporter expression, indicative of increased placental permeability [20].

Following the initial reports, several studies confirmed the presence of MPs and NPs in human placentas and suggested that detection frequency has risen over time. Weingrill et al. documented a progressive increase, with MPs detected in 60% of placentas in 2006, 90% in 2013, and 100% in 2021 [21]. Variations in MP composition were also observed, likely reflecting regional differences in plastic consumption and waste management practices [22,23]. As in animal models, human evidence now indicates that MPs and NPs can reach fetal compartments. Plastic particles have been identified in amniotic fluid, cord blood, and meconium—the latter showing the highest concentrations, with consistent detection in placenta and meconium but only occasional detection in amniotic fluid [24,25]. A recent study reported a positive correlation between MP abundance in placenta and cord blood (rs = 0.38, *p* < 0.05), suggesting that specific polymers—such as microplastic cellulose, polybutene isotactic, and polynorbornene—may preferentially cross the placental barrier. Plastic additives behave similarly; bisphenol-A (BPA) and phthalates have been detected in amniotic fluid, placental tissue, and maternal and cord blood, confirming their capacity for fetal exposure [26,27]. Nonetheless, substantial variability in MP/NP type and concentration has been reported across studies [28], indicating that exposure is influenced by multiple factors, including lifestyle, environment, and product use. At present, it remains unclear which particle sizes or polymers pose the highest risk, or how plastic-associated contaminants and additives transported by MPs and NPs contribute to fetal harm.

### 3.2. After Birth

Although exposure to MPs and NPs is widespread across the general population, infants and toddlers experience disproportionately higher exposure due to behavioral and physiological characteristics. Frequent hand-to-mouth behavior, crawling, and close contact with household dust, toys, and synthetic textiles increase ingestion and inhalation, while immature metabolic and excretory systems may alter particle absorption and clearance. One study estimated daily ingestion of polyethylene terephthalate (PET) and polycarbonate (PC) MPs from indoor dust at 6600 and 41 ng/kg/day in adults versus 120,000 and 750 ng/kg/day in infants [29]. Similar findings were reported for dietary exposure, where one-year-old infants ingested 83,000 ng/kg/day of PET and 860 ng/kg/day of PC compared with 5800 and 200 ng/kg/day in adults [30]. Correspondingly, infants excreted far higher fecal concentrations of PET and PC MPs—up to ten times adult levels (PET: 5700–82,000 ng/g vs. 2200–16,000 ng/g; PC: 49–2100 ng/g vs. 37–620 ng/g) [30].

Major postnatal sources of MPs and NPs include food, feeding equipment, and toys. MPs have been detected in breast milk, though at relatively low concentrations (<2.72 particles/g) [31,32]. In contrast, formula milk contains significantly more particles (up to 11.0 particles/g), due to MPs and NPs originating from powdered formula, water, and food-preparation utensils [33]. Feeding items such as plastic bottles, sippy cups, and coated cups can release millions of particles daily, especially when heated. For example, PE-coated paper cups, PP cups, and PS cups released 675–5984, 781–4951, and 838–5215 particles/L, respectively, after exposure to 95 °C water for 20 min [34]. Toys further contribute through mechanical abrasion during chewing and handling [35].

In older children and adolescents, diet remains a dominant source of exposure. Fadare et al. found 12 ± 5.12 mg of MPs in round food containers, 38 ± 5.29 mg in rectangular containers, and 3 ± 1.13 mg in disposable cups [36]. Bai et al. reported an average of 639 MP items/kg in take-out meals, with rice containing the most and coffee the least. Individuals consuming take-out one to two times weekly may ingest 170–638 particles per week [37]. Airborne exposure is also relevant. Torres-Agulló et al. found higher indoor MP concentrations in classrooms (21.8 ± 16.3 ng/m^3^) than outdoors (13.4 ± 13.6 ng/m^3^), with estimated inhalation exposure of 1.57 ± 0.93 ng/kg/day in children, along with elevated airborne phthalates [38].

For clinicians, these exposure profiles are especially relevant because infants and toddlers represent the age group most easily modifiable through preventive counseling. Avoiding the use of plastic feeding bottles with hot liquids, minimizing household dust exposure in children with asthma, and educating pregnant women on reducing contact with plastic food packaging are practical and low-cost measures that may decrease early-life intake and inhalation. Incorporating brief questions on diet, feeding practices, and household materials into routine pediatric and prenatal visits could therefore help identify children at higher exposure risk.

## 4. Toxicity Mechanisms of Plastic Particles and Plastic Additives

The toxic effects of MPs and NPs are driven by multiple biological pathways, including oxidative stress, inflammation, apoptosis, immune dysregulation, and genotoxicity. Plastic-associated chemicals—such as bisphenols, phthalates, and other additives incorporated during manufacturing—further amplify these mechanisms. These processes are interconnected and can act synergistically, increasing the likelihood of cellular injury from even a single agent [39]. Numerous studies support these observations [14], and the most representative examples are summarized below.

Exposure to MPs and NPs promotes excessive production of reactive oxygen species (ROS), including superoxide anions and hydrogen peroxide. At the same time, MPs impair endogenous antioxidant defenses by reducing the activity of superoxide dismutase, catalase, and glutathione, thereby disrupting cellular redox balance and triggering oxidative stress–mediated damage [40]. Several studies have confirmed this mechanism [41,42,43]. For instance, Palaniappan et al. reported increased expression of the superoxide dismutase 3 gene and reduced cell viability in murine fibroblast and canine kidney epithelial cells exposed to polyethylene (PE) and polystyrene (PS) MPs [44]. Similarly, Lim et al. demonstrated that environmentally relevant concentrations of NPs (0.0125 mg/mL) caused mitochondrial dysfunction and impaired respiratory activity in human liver and lung cells [45].

Inflammation arises because MPs and NPs are perceived as foreign particles capable of activating innate immune responses. This leads to persistent inflammation marked by elevated cytokines—including IL-6, IL-8, IL-10, and IL-1β—and activation of nuclear factor kappa-light-chain-enhancer of activated B cells (NF-κB), combined with inefficient macrophage clearance of ingested plastic particles. In parallel, MPs can induce gut dysbiosis, disrupting the balance between helper T cells (Th) and regulatory T cells (Tregs) [46], while also altering dendritic-cell function, reducing immune tolerance, and potentially contributing to autoimmunity and carcinogenesis [47].

Apoptosis represents another key toxicity mechanism. Numerous studies document the activation of programmed cell-death pathways following MP or NP exposure. Li et al. demonstrated that PS-MPs disrupt glycolytic pathways in hepatocytes, ultimately inducing apoptosis [48]. Umamaheswari et al. reported increased expression of apoptosis-related genes (TNF, p53, casp3b, gadd45ba, and ptgs2a) in zebrafish exposed to PS-MPs, accompanied by tissue alterations in the gills [48]. Likewise, Kwon et al. observed that microglial phagocytosis of 0.2–2 μm PS-MPs in human HMC-3 cells induced morphological changes and increased apoptotic markers such as BAX, cleaved PARP, and caspases 3 and 8 [49].

Regarding genotoxicity, MPs and NPs can directly interact with chromosomes, DNA, and nuclear proteins, or indirectly cause DNA damage through ROS production. Experimental evidence is abundant: comet-assay tail intensity in PS-exposed zebrafish heart tissue was shown to be 100-fold greater than in controls [50], and MP exposure increased micronuclei, nucleoplasmic bridges, and nuclear buds in human lymphocytes [51]. Even low doses have induced erythrocyte nuclear abnormalities in Crucian carp, suggesting persistent DNA injury during cell proliferation unless offset by apoptosis [52]. Moreover, prenatal MP exposure may influence genomic stability by shortening telomeres. Higher levels of polyvinyl chloride (PVC) and polybutylene succinate (PBS) correlate with shorter telomeres in cord blood, while polypropylene (PP) shows the strongest association in placental tissue. Telomere shortening—linked to impaired fetal growth and preterm birth—reflects progressive chromosomal instability with long-term implications [53].

Bisphenols and phthalates are major contributors to plastic-related toxicity. Bisphenols act as endocrine-disrupting chemicals (EDCs) with estrogen-mimicking and anti-androgenic properties, binding to estrogen receptors and other nuclear receptors. Their effects include oxidative stress, mitochondrial dysfunction, altered signaling pathways, and apoptosis, with particular relevance for reproductive tissues [54]. Phthalates exert similar endocrine-disrupting actions, interfering with hormonal regulation and affecting multiple organ systems [55,56].

Table 1 summarizes the toxicity mechanisms of microplastics, nanoplastics, and plastic additives.

## 5. Toxic Effects of Plastic Exposure in Pediatrics

### 5.1. Impact on Reproductive System and Reduced Fertility

Extensive experimental research across multiple animal species, including mammals and aquatic organisms, indicates that exposure to MPs and NPs can adversely affect reproductive health [57,58,59]. Additionally, MPs and NPs may serve as vectors for hazardous plastic additives (e.g., bisphenols and phthalates), further amplifying their potential to disrupt reproductive function. Notably, adverse outcomes have been observed even at low exposure levels and over short durations, with evidence suggesting that female reproductive systems may be more susceptible than male counterparts [60].

In females, PS-MP exposure has been shown to impair fertility across animal models. In fish, Wang et al. demonstrated that exposure to 2, 20, and 200 μg/L of PS-MPs for 60 days delayed gonadal maturation and reduced fecundity in Oryzias melastigma [61]. MPs also disrupted the hypothalamic–pituitary–gonadal (HPG) axis by downregulating steroidogenic genes and reducing circulating 17β-estradiol and testosterone. Parental exposure further resulted in delayed incubation, reduced hatching rates, and impaired offspring growth. Similarly, in rats, four estrous cycles of exposure to 5 μm PS-MPs resulted in reduced ovarian weight, altered folliculogenesis, shortened estrous cycles, and decreased estradiol levels, along with cytoskeletal protein dysregulation [62]. In mice, MP ingestion reduced oocyte maturation and fertilization, increased oocyte ROS levels, and induced mitochondrial dysfunction and apoptosis [63]. DNA damage, altered chromosome morphology, and reduced expression of actin and Juno further suggested impaired oocyte–sperm fusion.

In males, MPs and NPs can traverse the blood–testis barrier, accumulate in testicular tissue, and induce oxidative stress and inflammation, leading to sperm abnormalities, testosterone reduction, and impaired fertility through HPG-axis disruption [64,65,66]. Plastic additives can exacerbate these effects. BPA and phthalates interfere with testicular development by altering germ-cell differentiation, Sertoli cell maturation, and Leydig cell steroidogenesis during critical developmental windows [67].

Human studies support these experimental observations. Montano et al. detected MPs < 10 μm in 14 of 18 follicular fluid samples (average 2191 particles/mL), with a significant correlation between MP abundance and FSH levels [68]. In males, MPs have been found in testicular tissue and semen, with polymer profiles varying by matrix—PE and PVC predominating in semen, and PS in testis [69,70]. More recently, Zhang et al. associated polytetrafluoroethylene (PTFE) exposure with decreased semen quality in 113 adult males, showing reductions in sperm count, concentration, and motility, and demonstrating that cumulative MP exposure worsened semen parameters in a dose-dependent manner [71].

Clinically, the most relevant reproductive effects are attributed to endocrine-disrupting additives, especially BPA and phthalates. Prenatal exposure alters thyroid and sex hormone levels and vitamin D metabolism, increasing risks of maternal complications (e.g., preeclampsia, dysglycemia) and pediatric outcomes such as cryptorchidism, hypospadias, and reduced anogenital distance (AGD). Studies link phthalate exposure with altered pubertal timing, including earlier menarche and premature adrenarche [72,73]. Shortened AGD—one of the best-characterized biomarkers of fetal endocrine disruption—has been repeatedly associated with elevated maternal urinary phthalates [74,75,76,77,78,79,80] and is also observed in girls with endometriosis or polycystic ovary syndrome [81]. AGD correlates with adult sperm count and fertility potential [82,83].

However, findings are not universally consistent. Some studies reported no association between phthalate exposure and AGD [84,85], and research on cryptorchidism and hypospadias has yielded conflicting results [86,87]. Variability in study design, exposure timing, toxicant mixtures, sex-specific susceptibility, and genetic modifiers likely explains these discrepancies. Nonetheless, the parallel rise in global plastic production and male infertility between 1990 and 2019 suggests a concerning trend [88]. Until clearer evidence emerges, reducing exposure to phthalates and BPA during pregnancy remains a prudent preventive measure.

### 5.2. The Effects of Plastics on Children

From a clinical perspective, the disorders associated with MPs and additive exposure (Table 2) overlap with many of the most common reasons for pediatric consultation, including recurrent wheeze, behavioral and attention concerns, altered growth patterns, and early pubertal signs.

Because these conditions are often multifactorial, potential environmental contributors should be considered during clinical evaluation. Including a brief environmental history—covering feeding equipment, dietary packaging, indoor air quality, and parental occupational exposure—may support earlier recognition of children at risk and guide tailored prevention during critical developmental windows.

Beyond reproductive toxicity, studies demonstrate that prenatal and early-life exposure to plastics can adversely affect multiple organ systems. Animal experiments show that MPs and NPs accumulate in fetal tissues and contribute to growth restriction, metabolic dysfunction, and organ-specific damage in the liver, lungs, kidneys, heart, and brain [105]. The central nervous system appears particularly vulnerable: MP exposure has been linked to reduced cortical thickness, altered neuronal migration, abnormal neurogenesis, and behavioral phenotypes such as anxiety, memory impairment, and autism-like traits in offspring [89,95,96,106,107,108,109,110,111,112,113,114]. These outcomes vary by particle size, surface charge, and additive composition, and the critical exposure windows remain under investigation.

Emerging human evidence aligns with these findings. MPs were identified in all placentas from IUGR pregnancies in one study, with higher MP loads strongly correlating with reduced birth weight, length, head circumference, and Apgar scores [89]. Prenatal phthalate exposure has been repeatedly associated with neurodevelopmental effects—including impaired motor and memory skills, delayed language acquisition, autism traits, and ADHD-like behaviors. DEHP, DnBP, DMP, DEP, MiBP, and MEP are the most frequently implicated phthalates [90,95,96]. Behavioral studies further link MiBP exposure with aggression and rule-breaking behavior [97] and demonstrate sex-specific vulnerability, with boys more affected than girls [97,98]. BPA exposure shows similar associations, including preterm birth, reduced fetal growth, and smaller head circumference [91,92,93,94].

Plastic additives have also been implicated in respiratory and allergic diseases. Meta-analyses and cohort studies report associations between prenatal phthalate exposure and increased risks of asthma, wheeze, rhinitis, and eczema, with BBzP and DEHP showing the strongest correlations [99,100,101]. In metabolic pathways, prenatal phthalate exposure has been linked to childhood obesity, dyslipidemia, and impaired glucose metabolism [102,103,104]. Finally, there is growing concern that older children and adolescents may develop long-term conditions resembling those observed in exposed adults, including cancer, cardiovascular disease, respiratory dysfunction, and renal disorders [115,116,117,118].

Despite many positive associations, conflicting data exist. Some meta-analyses found no clear relationship between phthalate exposure and fetal growth [119], and studies on asthma and obesity have yielded mixed results [120,121]. These discrepancies likely reflect differences in exposure measurement, mixtures of toxicants, age at assessment, and population characteristics. Nonetheless, the replacement of DEHP with alternative plasticizers that proved equally or more harmful [122] illustrates the urgent need for more rigorous mechanistic and epidemiological research. Clarifying the specific risks posed by each plastic component is essential for protecting children’s health and guiding safer regulatory policies.

## 6. Conclusions

Available evidence indicates that children—particularly those exposed to plastic particles and additives during fetal life—are highly vulnerable to adverse health effects.

Plastic exposure may impair fetal growth, shorten pregnancy duration, and alter sexual development. It can also disrupt neurodevelopment, delay pubertal maturation, and increase the risk of allergic diseases, including asthma, as well as metabolic disorders such as obesity and diabetes. Despite these concerns, current research has not yet defined which plastic components are most detrimental, nor established exposure thresholds, critical windows of vulnerability, or the long-term persistence of health risks. Pediatricians and obstetric providers should consider environmental plastic exposure when evaluating disorders of growth, development, and endocrine function, and incorporate preventive counseling into routine care. Early-life guidance on reducing avoidable exposure may represent a simple and actionable public-health tool while awaiting stronger regulatory action. Further high-quality mechanistic and epidemiological studies are urgently needed. In the interim, stricter regulatory measures to limit nonessential plastic use—especially in low-income communities disproportionately affected by pollution—are warranted. Education of families and healthcare providers, public awareness initiatives, and transparent product ecolabelling may help reduce exposure. Ultimately, meaningful progress will require replacing harmful plastics with safer materials and adopting policies that prioritize child health and environmental protection.

## Figures and Tables

**Table 1:** Toxicity mechanisms of microplastics, nanoplastics, and plastic additives.

Mechanism	Description of Biological Effects	Representative Evidence	References
**Oxidative stress**	MPs/NPs increase ROS (e.g., superoxide, H_2_O_2_) and reduce antioxidant defenses (SOD, CAT, GSH), leading to cellular injury	ROS overproduction, mitochondrial dysfunction, and reduced cell viability observed in exposed animal and human cell models	[42,43,44,45,46,47]
**Inflammation**	Activation of cytokines (IL-6, IL-8, IL-10, IL-1β) and NF-κB, with impaired macrophage clearance and dysbiosis-induced immune activation	Chronic inflammation and microbiota imbalance demonstrated in experimental models	[48,49]
**Apoptosis**	MPs/NPs activate intrinsic and extrinsic apoptotic pathways, increasing caspases and pro-apoptotic markers	Altered glycolysis, mitochondrial damage, and up-regulated TNF, p53, casp3b, gadd45ba, and ptgs2a	[50,51,52]
**Genotoxicity**	Direct DNA interaction or indirect ROS-mediated damage causing micronuclei, DNA strand breaks, and chromosomal instability	Increased comet-tail DNA, nuclear abnormalities, and structural DNA damage in exposed cells and animals	[53,54,55]
**Telomere shortening**	MPs/NPs accelerate telomere erosion, compromising chromosome stability	Shorter cord-blood and placental telomeres associated with PVC, PBS, and PP exposure	[56]
**Endocrine disruption (Bisphenols, Phthalates)**	Bisphenols mimic/block hormones via estrogen/nuclear receptors; phthalates disrupt endocrine signaling	Oxidative stress, mitochondrial dysfunction, and reproductive-system interference	[57,58,59]

MPs = microplastics; NPs = nanoplastics; ROS = reactive oxygen species; SOD = superoxide dismutase; CAT = catalase; GSH = glutathione; IL = interleukin; NF-κB = nuclear factor kappa-light-chain-enhancer of activated B cells; TNF = tumor necrosis factor; PVC = polyvinyl chloride; PBS = polybutylene succinate; PP = polypropylene.

**Table 2:** Pediatric Health Effects of Plastic Exposure (MPs, NPs, and Additives).

System/Outcome	Main Health Effects in Children	Evidence Type	Key References
**Fetal growth & birth outcomes**	IUGR, reduced birth weight, prematurity	Human + Animal	[89,90,91,92,93,94]
**Neurodevelopment**	↓ IQ, memory deficits, ADHD traits, autism-like behaviors	Human + Animal	[90,91,92,93,94,95,96,97,98,99,100,101]
**Respiratory & allergy**	Asthma, wheeze, rhinitis, eczema	Human	[99,100,101]
**Metabolic effects**	Obesity, ↑ triglycerides, glucose dysregulation	Human + Animal	[102,103,104]
**Reproductive system**	Cryptorchidism, altered AGD, fertility impairment	Human + Animal	[57,58,59,60,61,62,63,68,69,70,71,72,73,74,75,76,77,78,79,80,81,82,83]
**Systemic/Other**	Inflammation, oxidative stress, multi-organ toxicity	Mostly animal	[89,95,96,105,106,107,108,109,110,111,112,113,114]

IUGR = intrauterine growth restriction; IQ = intelligence quotient; ADHD = attention-deficit/hyperactivity disorder; AGD = anogenital distance; MPs = microplastics; NPs = nanoplastics.

## Data Availability

All the available data are included in the manuscript.
